# G2019S *LRRK2* Mutation Enhances MPP^+^-Induced Inflammation of Human Induced Pluripotent Stem Cells-Differentiated Dopaminergic Neurons

**DOI:** 10.3389/fnins.2022.947927

**Published:** 2022-07-06

**Authors:** Ying Chen, Qing Yin, Xiao-Yu Cheng, Jin-Ru Zhang, Hong Jin, Kai Li, Cheng-Jie Mao, Fen Wang, Hong-Zhe Bei, Chun-Feng Liu

**Affiliations:** ^1^Department of Neurology and Clinical Research Center of Neurological Disease, The Second Affiliated Hospital of Soochow University, Suzhou, China; ^2^Baotou Medical College, Inner Mongolia University of Science and Technology, Baotou, China; ^3^Department of Neurology, The Third Affiliated Hospital of Inner Mongolia Medical University, Baotou, China; ^4^Jiangsu Key Laboratory of Neuropsychiatric Diseases and Institute of Neuroscience, Soochow University, Suzhou, China

**Keywords:** G2019S *LRRK2* mutation, Parkinson’s disease, induced pluripotent stem cells, MPP^+^, inflammation

## Abstract

Induced pluripotent stem cells (iPSCs) offer an unprecedented opportunity to mimic human diseases of related cell types, but it is unclear whether they can successfully mimic age-related diseases such as Parkinson’s disease (PD). We generated iPSCs lines from three patients with familial PD associated with the G2019S mutation in the *LRRK2* gene and one age-matched healthy individual (control). During long-term culture, dopaminergic (DA) neurons differentiated from iPSCs of G2019S *LRRK2* PD patients exhibited morphological changes, including a reduced number of neurites and neurite arborization, which were not evident in DA neurons differentiated from control iPSCs. To mimic PD pathology *in vitro*, we used 1-methyl-4-phenylpyridium (MPP^+^) to damage DA neurons and found that DA neurons differentiated from patients with G2019S *LRRK2* mutation significantly reduced the survival rate and increased apoptosis compared with the controls. We also found that the mRNA level of inflammatory factors [interleukin (IL)-1β, tumor necrosis factor-α, cyclooxygenase-2, IL-6, and inducible NO synthase] with G2019S *LRRK2* mutation were higher than control group after exposure to MPP^+^. Our study provides an *in vitro* model based on iPSCs that captures the patients’ genetic complexity and investigates the pathogenesis of familial PD cases in a disease-associated cell type.

## Introduction

Parkinson’s disease (PD) is the second most common neurodegenerative disease, and is characterized by progressive loss of dopaminergic (DA) neurons in the substantia nigra pars compacta (SNpc) (Reich and Savitt, 2019). The defining pathological feature of PD is abnormal accumulation and aggregation of Lewy bodies (LBs) composed of the protein α-synuclein (α-syn) (Goedert et al., 2013; Flagmeier et al., 2016). About 90% of PD cases are sporadic, while 10% show a single-gene form of the disease. Pathogenic mutations in *LRRK2* and *SNCA* genes have been identified and associated with PD. *LRRK2* mutations cause autosomal dominant inheritance, and their penetrance increases with age (Kachergus et al., 2005). The pathogenic mechanisms responsible for neurodegeneration in PD are unclear, mainly due to the lack of suitable animal genetic models. Therefore, there is an urgent need to develop reliable experimental models that mimic key neuropathological features of PD. More recently, induced pluripotent stem cells (iPSCs) have been used to model human disease. Specifically, iPSCs are able to differentiate into multiple different lineages, are easy to expand, easy to obtain, and do not require the destruction of embryos, reducing the ethical issues and criticisms associated with their use in research. In addition, the isolation of cells from patients opens the door to the potential development of patient-specific disease models and personalized medicine applications, which will be discussed in more detail later (Gonzalez et al., 2011; Smith et al., 2017).

The toxin 1-methyl-4-phenyl-1,2,3,6-tetrahydropyridine (MPTP) (Langston et al., 1983) induces selective death of SNpc neurons in human and experimental animal models (Gerlach and Riederer, 1996), and animal phenotypes similar to the clinical features observed in PD patients. MPTP is smoothly transported across the blood–brain barrier to lysosomes in striatum or nigra pars astrocytes, where it is cleaved by the enzyme monoamine oxidase B into toxic metabolite 1-methyl-4-phenylpyridium (MPP^+^) (Meredith and Rademacher, 2011). MPP^+^ is actively transported into the mitochondria of DA neurons via the dopamine transporter, and combines with NADH dehydrogenase (complex I) in the mitochondrial electron transport chain, leading to mitochondrial dysfunction by inhibition of mitochondrial respiration, rapid decrease in ATP content, or accumulation of α-syn (Subramaniam and Chesselet, 2013; Marti et al., 2017). DA neurons are terminally differentiated cells, and thus are highly susceptible to oxidative stress, which eventually leads to their loss in the nigra pars.

Pathogenic mutations in *LRRK2* and *SNCA* have been associated with PD, while mutations in four genes (*Parkin*, *DJ-1*, *PINK1*, and *ATP13A2*) cause early-onset parkinsonism (Lees et al., 2009). In particular, *LRRK2* mutations cause autosomal dominant, late-onset familial PD, whose clinical and pathological features are hard to distinguish from the common, sporadic form of PD (Paisan-Ruiz et al., 2004). While over 50 variants have been identified throughout the different *LRRK2* domains in PD patients, the mutation G2019S is regarded as the most common cause of dominant familial PD (Cookson, 2010; Sanchez-Danes et al., 2012). In the early stage, our research group identified 18 rare variants in 27 PD patients with *LRRK2* mutation by target region sequencing and exome sequencing of 296 PD patients. Furthermore, these PD patients with *LRRK2* mutation are easier to generate motor fluctuations and non-motor symptoms (Zhang et al., 2018).

Although the etiology of PD is unclear, it seems likely that inflammation plays an important role. Microglial activation in the SNpc and other affected regions, as well as elevated levels of proinflammatory cytokines, has been detected in the postmortem brain of PD patients (Sawada et al., 2006). High levels of pro-inflammatory cytokines, including interleukin (IL)-1β, tumor necrosis factor (TNF)-α and IL-6, which are key signaling molecules for immune activation, have been found in the brain, serum and cerebrospinal fluid in PD patients. These pro-inflammatory cytokines in the brain are mainly produced by microglia and other infiltrating peripheral myeloid cells (Mogi et al., 1994a,b; Hu et al., 2015). The association between PD and increased inflammation has been well established (Han et al., 2019). Both men and women over the age of 65 years have increased serum IL-6, TNF-α, and IL-1β levels (Pedersen et al., 2003; Ferrucci et al., 2005). IL-1β, a major regulator of neuroinflammation (Basu et al., 2004) is mainly produced by activated inflammatory cells of the myeloid lineage and it contributes significantly to cellular activation and cytokine production (Giuliani et al., 2017). IL-1β plays a key role in the pathogenesis of both central and peripheral nervous system diseases (Dinarello et al., 2012; Tanaka et al., 2013).

Taken together, our iPSC-based PD model provides a valuable tool for studying the pathogenesis of PD. The altered inflammation level is consistent with the fact that PD patients with G2019S *LRRK2* mutation are more vulnerable to neurotoxins related to PD, such as MPP^+^.

## Materials and Methods

### Generation and Passaging of Human Induced Pluripotent Stem Cells

Human blood samples were collected from personal sodium citrate collection tubes approved by the Second Affiliated Hospital of Soochow University Clinical Trial Laboratory Service and the Ethics Committees. Blood samples were isolated by gradient centrifugation of peripheral blood monocytes (PBMCs). IPSCs from healthy donors and patients with PD were generated using Sendai virus vectors as previously reported. Informed consent was obtained from all individuals. Cells that had normal karyotypes (46, XX or 46, XY) and no mycoplasma contamination were detected. Whole-exome analyses of three patients were performed at the Second Affiliated Hospital of Soochow University, and we did not detect any other PD-related genes mutations except *LRRK2* G2019S in the following: *Parkin*, *GBA*, *SNCA*, *PINK1*, *DJ-1*, *UCHL1*, *ATP13A2*, *GIGYF2*, *Omi/HTRA2*, *PLA2G6*, *FBXO7*, *VPS35*, *EIF4G1*, *DNAJC6*, *SYNJ1*, *CHCHD2*, and *RAB39 B* (Zhang et al., 2018). The human iPSCs were cultured in a feeder-free condition in Essential 8™ Basal Medium (Thermo Fisher Scientific). The medium was changed daily and the cells were passaged every 5–7 days, depending on the confluence of the plates. For passage of human iPSCs, cells were washed with PBS and incubated with dispersing enzyme (Invitrogen) for 45 min. Colonies were collected in Falcon tubes containing iPSC growth medium and allowed to settle for 30 s. The supernatant containing residual dispase was removed and the colonies were washed once with iPSC medium. After the colonies were allowed to settle again, the supernatant was removed. Finally, they were mechanically broken down and plated onto Matrigel-coated plates. Cells were passaged in the desired proportion every 5–7 days (depending on the confluence of the plates).

### Differentiation of Dopaminergic Neurons

The neutralization of human iPSCs induced by dual SMAD inhibition allowed rosette neural stem cell (NSC) with DA potential. Cells were incubated in the neural induction media (consisting of DMEF/F12, NEAA 1×, sodium pyruvate 1×, GlutaMax 1×, 2-mercaptoethaol 1×, and penicillin streptomycin 1×) with 500 ng/ml Noggin and 10 μM SB431542 for 4 days. The cells were cultured for a further 5 days in the DA neuron induction media with 500 ng/ml Noggin and 200 ng/ml Sonic hedgehog (SHH), provided by N2. Cells were cultured in DA neuron induction media for 5 days with brain-derived neurotrophic factor (BDNF, 20 ng/ml; R&D Systems), SHH (200 ng/ml, Sigma), L-ascorbic acid (L-AA, 200 μM; Tocris), and fibroblast growth factor 8b (FGF8b, 100 ng/ml; Sigma). The medium was changed every other day. BDNF (20 ng/ml, R&D Systems), glial-derived neurotrophic factor (20 ng/ml; R&D Systems), transforming growth factor β3 (1 μM; R&D Systems), L-AA (200 μM; Tocris) and cAMP (1 mM; Sigma) were exposure to N2 medium for an additional 14 days and DA neurons were further matured. The matured DA neurons exhibited typical morphology, and immunocytochemical responses for doublecortin (DCX) and tyrosine hydroxylase (TH) confirmed the identification of cells.

### Flow Cytometry

DA neurons from human iPSCs were collected by centrifugation with ice-cold PBS and resuspended in 500 μl 1 × Binding Buffer. Then, 5 μl of Annexin V-FITC and 10 μl PI were added to each tube. After vortex gently, the mixture was incubated at room temperature in the dark for 5 min. On a flow cytometer, Annexin V-FITC (Ex = 488 nm; Em = 530 nm) was detected by a FITC detection channel, and PI was detected by a PE detection channel.

### Cell Counting Kit-8 Assay

Cell Counting Kit-8 (CCK-8) (Dojindo Molecular Technologies) was used to measure cell viability. Cell suspension were inoculated at 100 μl/well into 96-well plates at a density of 10^4^ cells/well. After stimulation, we removed the culture medium and cells were washed twice by prewarmed PBS. CCK-8 solution was added to each well at a final concentration of 10%, and incubated for 2 h under 5% CO_2_ at 37^°^C. OD at 450 nm was measured by a Microplate Reader (Bio-Rad).

### Immunocytochemistry

DA neurons were seeded on 20-mm diameter glass coverslips that were individually placed in each well of 12-well plates and fixed in 4% PFA for 10 min at room temperature. Cells were washed and permeabilized with 0.1% Triton X-100 and blocked in 10% BSA in PBS for 30 min before staining. The cells were incubated overnight at 4^°^C with the following primary antibodies: mouse anti-TH (1:1,000, ab129991; Abcam), rabbit anti-nestin (1:1,000, ab105389; Abcam), mouse anti-SOX2 (1:1,000, ab79351; Abcam), and mouse anti-DCX (1:2,000, ab18723; Abcam). The detection of target antigen was performed by species-appropriate Alexa Fluor 488 or 555-conjugated secondary antibodies. Cells were visualized and imaged by a Leica inverted fluorescence microscope.

### Western Blotting

Protein levels were assessed by western blotting. DA neurons were seeded at a density of 6 × 10^4^/cm^2^ in 35-mm dishes and were collected after reaching 90% confluence. Cells were lysed in lysis buffer consisting of 5 mM EDTA, 25 mM Tris, 150 mM sodium chloride and protease inhibitor cocktail (Sigma). We determined the protein concentrations of each sample in the supernatants using the bicinchoninic acid assay. Proteins (30 μg) were loaded on 10% SDS–PAGE. After transferring proteins to polyvinylidene difluoride (PVDF) membranes, the membranes were blocked in Tris-buffered saline with 5% milk powder for 1 h at room temperature. The PVDF membranes were incubated with the following primary antibodies: pSer129 α-syn (1:500, ab51253; Abcam), α-syn (1:1,000, ab138501; Abcam), anti-β-actin (1:5,000, ab6276; Abcam), overnight at 4^°^C. The membranes were incubated with horseradish-peroxidase-conjugated secondary antibodies, and the proteins were visualized using ECL detection kits (GE Healthcare) on chemiluminescence (Clinx Science Instruments Co., Ltd.).

### RNA Extraction and RT-PCR

RT-PCR was used to measure the levels of TNF-α, IL-1β, COX-2, iNOS, and IL-6 in each group. Total RNA was extracted with TRIzol (Sigma-Aldrich) and cDNA was synthesized using a high-capacity cDNA reverse transcription kit (Thermo Scientific). Quantitative real-time PCR was performed on a 7,500 real-time PCR system (Applied Biosystems) with custom-designed primers using HiPi real-time PCR SYBR Green Master Mix and GAPDH for an internal control. The primers used are shown in Table 1. Cycling conditions were: initial denaturation for 3 min at 94^°^C, denaturation for 30 s at 94^°^C, annealing for 30 s at 60^°^C, and extension for 1 min at 72^°^C, followed by 40 cycles. The expression value of the target gene was normalized to GAPDH and quantified relative to the expression level of the control samples.

**TABLE 1:** PCR primers used in this study.

Probe	Orientation	Sequence
COX-2	Forward	AGCACTTCACGCATCAGTTTTTC
COX-2	Reverse	GCCTGAGTATCTTTGACTGTGGG
IL-1β	Forward	TTCCCTGCCCACAGACCTT
IL-1β	Reverse	CTTTTTTGCTGTGAGTCCCGG
IL-6	Forward	CCCCAGGAGAAGATTCCAAAGA
IL-6	Reverse	CAAACTCCAAAAGACCAGTGATGAT
TNF-α	Forward	TCTACTCCCAGGTCCTCTTCAAG
TNF-α	Reverse	GGAAGACCCCTCCCAGATAGA
iNOS	Forward	TCGTGGAGACGGGAAAGAAG
iNOS	Reverse	CCTGGGTCCTCTGGTCAAACT

### Statistical Analysis

GraphPad Prism 8 software was used for statistical analysis. One-way analysis of variance was applied for the assessment of differences among the graphs. Once the significance was found, the differences were further analyzed by Dunnett’s test. Data are presented as mean ± SEM, and *P* < 0.05 was considered statistically significant.

## Results

### Protocol for Fast and Efficient Production of Neural Stem Cells From Human Induced Pluripotent Stem Cells

**FIGURE 1:**
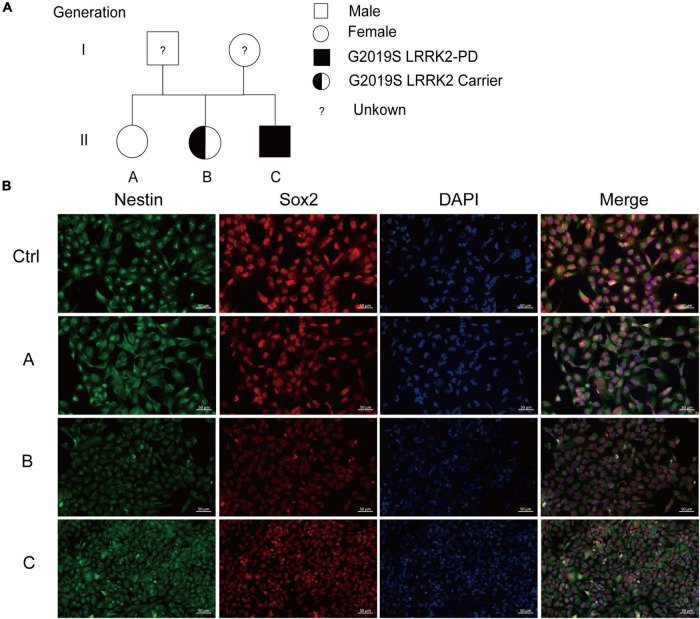
Generation and characterization of iPSCs-derived NSCs. **(A)** The genetic family diagram of G2019S *LRRK2* pedigree patients. Donor A did not carry the G2019S *LRRK2* mutation or had PD; donor B carried the G2019S *LRRK2* mutation but without PD; and donor C carried the G2019S *LRRK2* mutation and had PD. **(B)** Representative images show immunofluorescence staining for markers for iPSCs-derived NSCs (SOX2 in red, nestin in green). Nuclei counterstained with DAPI, shown in blue. Scale bars, 50 μm.

We collected blood from one healthy person and three *LRRK2* G2019S patients (the genetic family diagram is shown in Figure 1A) and held them at room temperature for 5 h before isolating PBMCs and reprogramming them into human iPSCs (Agu et al., 2015), and rapidly and efficiently generating DA neurons. This protocol was established using a human iPSCs line, and the first step in differentiation into DA neurons was the generation of NSCs. To this end, we cultured iPSCs in a neural induction medium for 10 days. We performed immunolabeling to examine the phenotype of newly generated NSCs, all expressing a typical combination of neuroectodermal markers, including the transcription factor SOX2, and the intermediate filament protein nestin. Immunofluorescent labeling supported the induction of neuronal differentiation, as demonstrated by reduced nestin and SOX2 expression in *LRRK2* G2019S mutation carriers compared to the normal individual (Figure 1B). The NSCs play an important role in the differentiation protocol, as these cells can be scaled up and frozen, hence allowing initiation of the differentiation prototype of all DA neurons.

### Effects of G2019S *LRRK2* Mutation on Differentiation of Neural Stem Cells Into Dopaminergic Neurons

**FIGURE 2:**
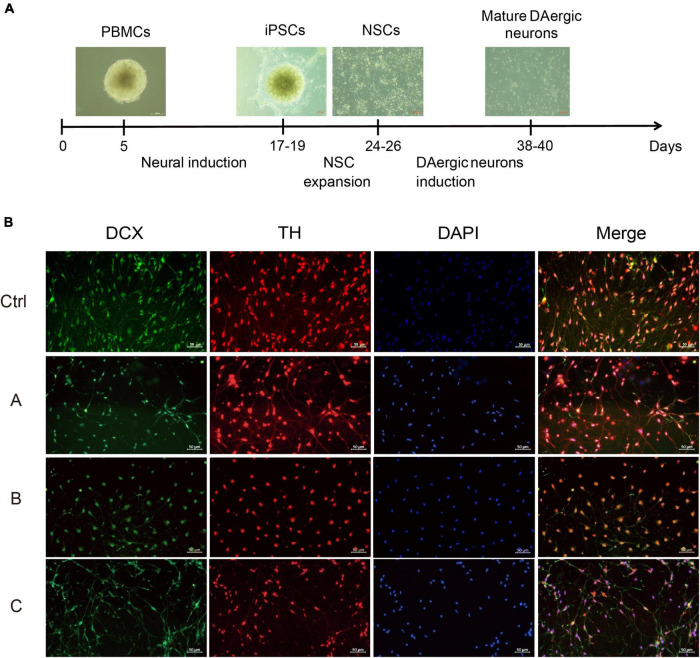
Differentiation and characterization of iPSCs-derived DA neurons. **(A)** Scheme showing the protocol for the generation of DA neurons. **(B)** DA neurons were analyzed by immunofluorescence for expression of DCX (green) and TH (red) at the end of the 38-day differentiation protocol. Nuclei counterstained with DAPI, shown in blue. Scale bars, 50 μm.

The next step was to generate DA neurons that show ventral midbrain characteristics by differentiation from NSCs (Figure 2A). DA neuron progenitors were cultured in the DA neuron differentiation medium. The progressive differentiation of these neurons into DA neurons was supported by increased expression of a combination of DA markers including TH and DCX, a protein specifically expressed by neural precursor cells and new neurons that can be used to identify early, immature neuron. Immunofluorescence showed that TH and DCX expression was reduced in G2019S *LRRK2* mutation carriers compared with the normal individual, suggesting a diminished ability to induce differentiation into DA neurons (Figure 2B).

### G2019S *LRRK2* Mutant Human Dopaminergic Neurons Are More Sensitive to 1-Methyl-4-Phenylpyridium^+^ Exposure

**FIGURE 3:**
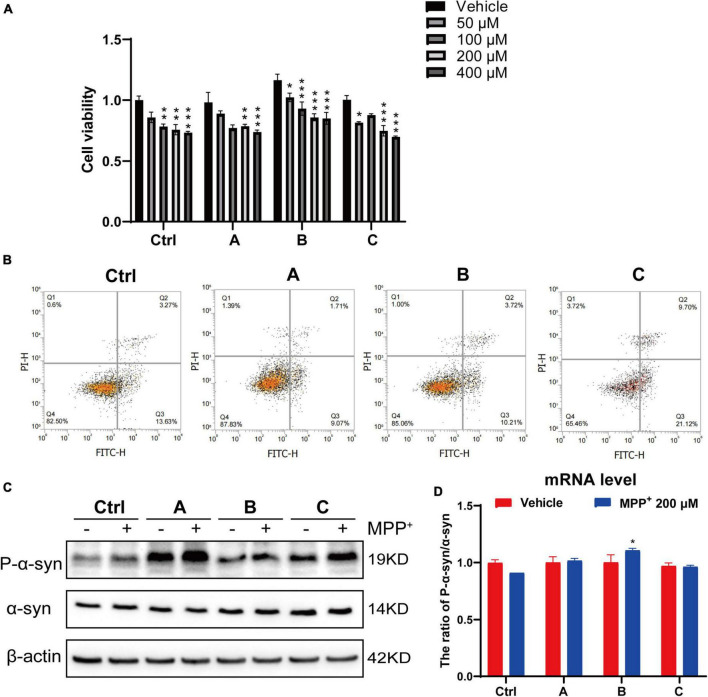
G2019S *LRRK2* human DA neurons were more sensitive to MPP^+^ exposure. **(A)** After iPSCs-derived DA neurons were exposed to different concentrations of MPP^+^ for 24 h, the cell viability was measured by CCK-8 assay. **(B)** Representative flow cytometry data of Ctrl and G2019S *LRRK2* mutant DA neurons labeled with Annexin V–FITC. **(C)** Representative blots probed control and G2019S *LRRK2* mutant DA neurons with antibodies against pSer129 α-syn (P-α-syn) and total α-syn. β-actin was used as an internal control. **(D)** Relative P-α-syn and total α-syn mRNA levels measured using quantitative RT-PCR analysis (*n* ≥ 3 samples/group). Data are expressed as mean ± SEM; *P < 0.05; **P < 0.01; ***P < 0.001.

MPP^+^ is a neurotoxin widely used to induce apoptotic cell death in experimental models of PD (Li et al., 2019; Yakhine-Diop et al., 2021). A dose-dependent study was conducted to assess the effect of MPP^+^ on the death of DA neurons. The DA neurons were incubated with different concentrations of MPP^+^ (0, 50, 100, 200, and 400 μM) for 24 h, and cell viability was measured by CCK8 assay. Cell viability was significantly decreased with increasing concentration of MPP^+^ (>100 μM) (Figure 3A). MPP^+^ at a concentration of 200 μM was used in the follow-up experiments. We observed that 200 μM MPP^+^ resulted in ∼24% cell death in the control, which increased to ∼27% in the G2019S *LRRK2* mutant group. Consistent with these results, flow cytometry (Figure 3B) showed that the early apoptosis rate of normal human DA neurons exposed to 200 μM MPP^+^ was 13.63, and 21.2% in G2019S *LRRK2* mutant DA neurons. MPP^+^ (200 μM) significantly upregulated phosphorylation of α-syn protein and mRNA expression in G2019S *LRRK2* mutant human DA neurons (Figures 3C,D). In conclusion, it is important to note that G2019S *LRRK2* mutant human DA neurons are already more sensitive than DA neurons without the mutation.

### G2019S *LRRK2* Mutant Human Dopaminergic Neurons Produce More Pro-inflammatory Factors Exposed to 1-Methyl-4-Phenylpyridium^+^

**FIGURE 4:**
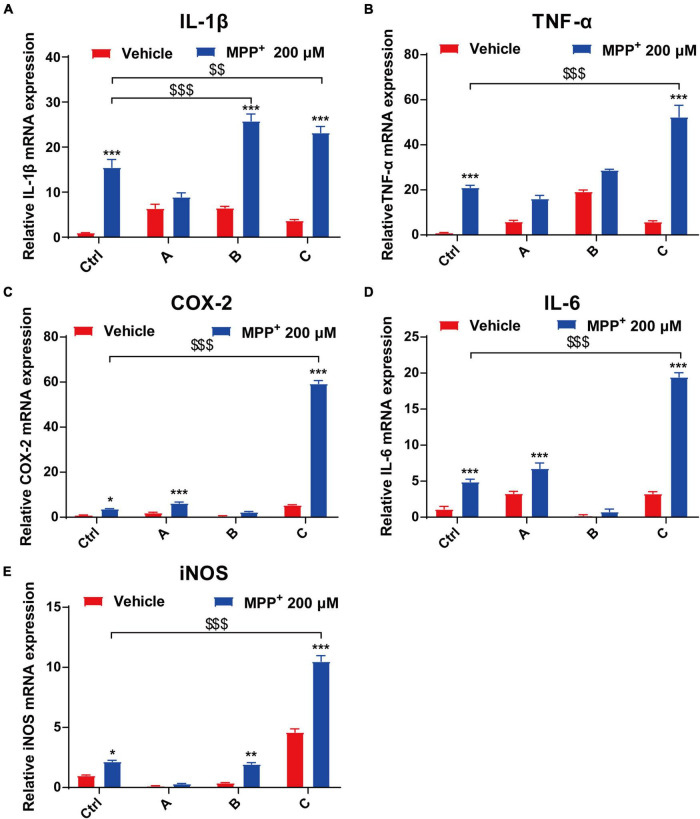
MPP^+^ increases pro-inflammatory factor levels in DA neurons with the G2019S *LRRK2* mutation. The iPSCs-derived DA neurons were exposed to 200 μM MPP^+^ for 24 h. **(A–E)** Data are presented as relative expression levels of IL-1β, TNF-α, COX-2, IL-6, and iNOS mRNA (normalized with respect to GAPDH) are mean ± SEM, n ≥ 3, *P < 0.05, **P < 0.01, ***P < 0.001, ^$$^*P* < 0.01 and ^$$$^*P* < 0.001.

In the brain, the pro-inflammatory cytokines TNF-α, IL-1β, IL-6, and COX-2 play important roles in a wide range of inflammatory responses to both acute and chronic conditions (Spooren et al., 2011; Kalb et al., 2021). We used quantitative PCR to analyze mRNA expression of IL-1β, TNF-α, COX-2, IL-6, and iNOS from DA neurons. After 24 h incubation with MPP^+^, the levels of the pro-inflammatory factors significantly increased. Moreover, there was a 16-fold increase in COX-2 in G2019S *LRRK2* mutant DA neurons compared to the control. Additionally, transcript levels of IL-1β and TNF-α in G2019S *LRRK2* mutant DA neurons were approximately twofold higher than control group (Figures 4A–E).

## Discussion

The etiopathogenesis of PD remains unclear. Although the majority of PD cases are idiopathic, the effects of mutations associated with familial PD, such as *SNCA*, *LRRK2*, *PARK2*, *PINK1*, and *GBA* remain the focus of current research (Zhang et al., 2021). There are still several obstacles to study PD despite the availability of mouse models and *in vitro* assays. First, there are no particularly reliable biomarkers available for preclinical diagnosis of PD. Second, the cause of DA neuronal death at the cellular level alone is largely unknown, and there is currently no effective therapeutic target. Third, the underlying pathology is not well understood in relevant disease models, because affected neurons cannot be obtained from PD patients (except for postmortem tissue of limited value), and animal studies are often inadequate to represent what happens in human disease. Hence, we and others already have that iPSCs technology, to some extent, may be able to reduce these limitations.

Since iPSCs can be produced from both skin fibroblasts and blood, and footprint-free and be differentiated *in vitro*, we generated a large number of neurons from families of PD patients with *LRRK2* gene mutations for mechanical disease modeling and drug screening. We described the generation and characterization of a panel of iPSC lines representing G2019S *LRRK2*-mutated PD patients and a healthy control. Next, we generated human DA neurons from iPSCs by established protocols and characterized them by expression of DCX and TH. Consistent with previous studies, our data showed that iPSCs with G2019S *LRRK2* mutation had a weaker ability to induce differentiation into DA neurons, which was mainly reflected in lower TH-positive neuron expression and immature neuronal precursor marker DCX in patients with G2019S *LRRK2* mutation compared to the healthy individual. However, under our modified protocol, it was difficult to maintain DA neurons in culture for > 40 days because cells tend to spontaneously separate and die. Besides, this method is limited to G2019S *LRRK2* mutant PD-iPSC lines, and there are no multiple subclones and controls.

At present, the relationship between environment and genes is well understood (Dardiotis et al., 2013). Neurotoxins such as MPP^+^ (Emborg et al., 2013; Kikuchi et al., 2017) and genetic mutations in *PARK* genes, such as *LRRK2* (West et al., 2005), have become major components of the origin of PD. Leucine-rich repeat kinase 2 (*LRRK2*) is involved in inflammation (Wallings and Tansey, 2019; Cabezudo et al., 2020; Oliveira et al., 2021). Thus, there is a link between inflammation and PD risk factors. Since iPSC lines derived from PD patients are reported to be more susceptible to oxidative stress (Nguyen et al., 2011), we applied MPP^+^ as a neurotoxin to induce DA neuronal damage to mimic PD pathology *in vitro*. Interestingly, we discovered that human DA neurons with the G2019S *LRRK2* mutation displayed a higher sensitivity to MPP^+^ exposure than the control individual did. In addition, we found that the transcriptome level of inflammatory factors in DA neurons with G2019S *LRRK2* mutation was significantly higher in PD patients than in the healthy mutation-free individual. Therefore, it is necessary to clarify the correlation between elevated levels of inflammatory factors and increased sensitivity of G2019S *LRRK2*-mutated DA neurons.

To our knowledge, neuroinflammation involves the activation of microglia and astrocytes to release inflammatory mediators within the brain, and the subsequent recruitment of peripheral immune cells (Montaner et al., 2010; Schain and Kreisl, 2017). In recent years, many studies have reported that chronic inflammation can lead to the degeneration of DA neurons and progression of PD (Gao and Hong, 2008; Spielman et al., 2016; Schain and Kreisl, 2017; Wang et al., 2020). Our previous study has verified abnormal activation of glial cells and highly expressed inflammatory factors such as IL-1β and TNF-α in MPTP-treated mice (Liu et al., 2020). In addition, the levels of inflammatory cytokines, especially TNF-α, IL-1β, IL-6, IL-8, and IFN-γ and large amounts of reactive oxygen species and nitrogen were elevated in the blood, cerebrospinal fluid, striatum and SN of PD animal models and patients (Mogi et al., 1994b,^1996^; Mantle and Lee, 2018). Besides, several studies have reported the dysregulation of inflammatory events by *LRRK2 in vivo*. Daher et al. (2015) reported increased activation of microglia in the SN of G2019S *LRRK2* transgenic rats following recombinant adeno-associated viral vector-mediated overexpression of α-syn. However, this increase in neuroinflammation could be abrogated by inhibiting LRRK2 activity (Daher et al., 2015). Recently, another study showed increased CD68 expression in microglia from G2019S *LRRK2* mice injected with recombinant α-syn fibrils, accompanied by increased expression of pro-inflammatory markers such as IL-6, TNF-α and C1qa and astroglia markers such as Vim, CD44 and Cxcl10 (Bieri et al., 2019). Importantly, we observed a significant elevated level of different inflammatory cytokines, such as TNF-α, IL-1β, IL-6, COX-2, and iNOS in human iPSC-differentiated DA neurons with *G2019S LRRK2* mutation. Taken together, LRRK2 is considered to be a pro-inflammatory agent in different neuroinflammatory animal models with increased kinase activity as a driver of inflammation (Cabezudo et al., 2020). Indeed, several studies have proposed potential immune-related roles for LRRK2. For example, altered inflammatory and degenerative phenotypes have been reported in *LRRK2*-mutant rodents following LPS challenge (Daher et al., 2014; Moehle et al., 2015). *In vitro* exposure to pathogens and inflammatory mediators such as IFN-γ, IFN-β, TNF-α, and IL-6 upregulates LRRK2 mRNA and protein levels in macrophages and white blood cells (Hakimi et al., 2011; Kuss et al., 2014). In addition, Toll-like receptor stimulation leads to phosphorylation, dimerization and membrane translocation of LRRK2, indicating activation of its function (Schapansky et al., 2014). Intriguingly, LRRK2 protein levels are elevated in B cells, T cells and CD14^+^ and CD16^+^ monocytes in PD patients compared with healthy controls. A vital role for *LRRK2* is supported by the IFN-γ response in infection and DA neuron loss. Further evidence comes from a recent study that has suggested that the *LRRK2* G2019S mutation makes DA neurons sensitive to IFN-γ by reducing AKT phosphorylation and inhibiting nuclear factor of activated T-cells (NFAT) nuclear shuttle, which in turn leads to shortened neurites (Panagiotakopoulou et al., 2020).

Moreover, the accumulation of α-syn protein is also implicated in *LRRK2* gain-of-function mutations (Tong and Shen, 2009; Rui et al., 2018). α-syn is a major component of LBs, aggregates of proteins that form in neurodegenerative disorders such as LB disease, Alzheimer’s disease and PD (Spillantini et al., 1998; Bayer et al., 1999; Schweighauser et al., 2020). Previous studies have demonstrated that overexpression of *LRRK2* G2019S protein can lead to abnormal accumulation of α-syn, either as a direct result of protein phosphorylation by *LRRK2* or indirectly increased *SNCA* gene expression or protein stabilization through an altered kinase signaling pathway (Novello et al., 2018; Bieri et al., 2019). Similarly, we observed a significant increase in pSer129 α-syn protein in G2019S mutant human DA neurons, which is consistent with previous reports.

In conclusion, the relationship between *LRRK2* mutations, accelerated α-syn pathology, and glial cells deserves further investigation. This information may help us better understand the interaction between environmental and genetic factors that may contribute to neuronal death in PD. Further work is needed to determine whether suppression of inflammatory factor levels reduces the sensitivity of neurons affected by the genetic mutation. Furthermore, this fact is critical for the development of future treatments aimed at reducing cellular mortality from exposure to certain environmental factors.

## Data Availability Statement

The original contributions presented in this study are included in the article/supplementary material, further inquiries can be directed to the corresponding author/s.

## Ethics Statement

The studies involving human participants were reviewed and approved by the Ethics Committee of the Second Affiliated Hospital of Soochow University. The patients/participants provided their written informed consent to participate in this study.

## Author Contributions

YC and C-FL conceived and designed the experiments. YC, QY, and X-YC performed the experiments. FW, C-JM, and YC analyzed the data. H-ZB and C-FL contributed to reagents, materials, and analysis tools. YC drafted the manuscript. C-FL revised the manuscript. All authors read and approved the final manuscript.

## Conflict of Interest

The authors declare that the research was conducted in the absence of any commercial or financial relationships that could be construed as a potential conflict of interest.

## Publisher’s Note

All claims expressed in this article are solely those of the authors and do not necessarily represent those of their affiliated organizations, or those of the publisher, the editors and the reviewers. Any product that may be evaluated in this article, or claim that may be made by its manufacturer, is not guaranteed or endorsed by the publisher.

## Abbreviations

α-syn: α-synuclein
BDNF: Brain-derived neurotrophic factor
CCK-8: Cell counting kit-8
DA neuron: Dopaminergic neuron
DCX: Doublecortin
iPSCs: Induced pluripotent stem cells
IL-1 β: Interleukin-1 β
LRRK2: Leucine-rich repeat kinase 2
LB: Lewy body
MPTP: 1-methyl-4-phenyl-1,2,3,6-tetrahydropyridine
MPP^+^: 1-methyl-4-phenylpyridium ion
mRNA: Messenger RNA
NSCs: Neural stem cells
PD: Parkinson’s disease
PBMCs: Peripheral blood mononuclear cells
PFA: Paraformaldehyde
PVDF: Polyvinylidene difluoride
SNpc: Substantia nigra pars compacta
TH: Tyrosine hydroxylase
TNF: Tumor necrosis factor.
